# From consortium design to bioaugmented filters: scalable yeast-based strategies for lead remediation in water systems

**DOI:** 10.3389/fmicb.2025.1647398

**Published:** 2025-09-10

**Authors:** Nikita Gupta, Sathiavelu Arunachalam

**Affiliations:** ^1^School of BioSciences and Technology, VIT, Vellore, Tamil Nadu, India; ^2^VIT School of Agricultural Innovations and Advanced Learning, VIT, Vellore, Tamil Nadu, India

**Keywords:** Cauvery river, consortium, heavy metal pollution, lead, yeast

## Abstract

Our study aimed to utilize the yeast consortium formed from the native heavy metal-resistant yeasts isolated from the River Cauvery to bioremediate lead. As an extension of the study, the same optimized consortium was further used to augment alginate-based filters to showcase an early proof of concept example that the biosorptive potential of yeast could improve the functionality of these filters. Three yeast strains native to the river Cauvery and highly resistant to heavy metal presence, viz. *Clavispora lusitaniae* (R4N2), *Candida tropicalis* (R2N4), and *Pichia kudriavzevii* (R1N8) were used to design a compatible and synergistic consortium for this study. For optimizing the performance of the consortium over so many independent variables, we took the help of a computational modelling approach, i.e., RSM (Response surface modelling), to narrow down the effective number of experiments. The Box–Behnken design (BBD) matrix within the RSM framework was used extensively in this study. For highlights, in single culture optimization: *Candida tropicalis* reached near-complete removal at pH 7, biomass 2 g, and Pb^2+^ 200 mg/L; *Clavispora lusitaniae* reached maximum removal (~100%) at pH 5.5–7.0, biomass dosage above 1.4 g, and Pb^2+^ concentrations of 120–200 mg/L; *Pichia kudriavzevii* performed best at pH 6.13, biomass 1.53 g/L, and Pb^2+^ concentration of 151.80 mg/L. For the 2-mix consortium (R2N4 + R4N2) removal efficiency over the optimized condition was 93.77% for 100 ppm and 52.42% at 200 ppm. For the 3-mix consortium, removal efficiency was 97.49% at 100 ppm and 52.11% at 200 ppm. The lead removal was further improved when we coupled the consortium with alginate gel slabs. At 100 ppm and 500 ppm, the 2-mix filter assembly removed 99.39 and 93.77% of the Lead, while the 3-mix filter assembly removed 99.97 and 95.19% of the Lead. Lead deposition on the filter surface and cells via biosorption was validated by SEM, FTIR, and EDX experiments. To conclude, our study shows that the bioaugmented filter allows for efficient removal of lead from water at lab-scale operations with further potential for scale-up and industrial usage in wastewater treatment.

## 1 Introduction

A huge amount of waste has been generated over several decades due to the rapid industrial and urban growth and development. These wastes comprise hydrocarbons, heavy metals, detergents, etc. It is well known that most of these wastes are byproducts of several anthropogenic and energy generation activities (Kulshreshtha et al., 2014). For heavy metal contamination, sometimes the source of pollution also includes natural leaching from soil rich in metal ore, for example, Iron contamination in the context of the river Cauvery. Most heavy metal contaminants in the environment are Cadmium, Lead, Chromium, Copper, Nickel, Cobalt, Iron, and Zinc. Many of these heavy metals, such as Lead, Cadmium, and Chromium, are well-known carcinogens and are toxic to human health and well-being (Xie, 2024). In recent years, there has been significant acknowledgement globally for the heavy metal contamination, among which Lead (Pb^2+^) is considered one of the most toxic HMs. Lead is persistent in the ecosystem and tends to bioaccumulate across various trophic levels in the food chain. Lead has its uses in several industrial processes, viz., mining, battery manufacturing, smelting, and paint/pigment production (Zhang et al., 2015). As a byproduct of these processes significant amount of lead often finds its way into the environment. The World Health Organization (WHO) & United States Environmental Protection Agency (USEPA) have several guidelines for the maximum tolerable limit for various HM in water. For lead in drinking water, 0.05 mg/l is the tolerable limit, which reflects the toxicity of this heavy metal. Prolonged exposure to higher levels of Lead is associated with neurological damage, developmental delays in kids, dysfunctioning of the kidneys, and other cardiovascular diseases (USEPA, 2000; Coeurdassier et al., 2004; CPCB, 2019). Hence, proper control over lead pollution in water sources is a critical and unmet need.

Conventional methods for heavy metal removal include chemical precipitation, ion exchange, membrane filtration, and adsorption. These methods, although effective, come with several limitations, viz., high operational cost, limited selectivity, generation of secondary contaminants, and need for a dedicated setup/infrastructure (Rahman and Singh, 2020). We know that chemical precipitation of heavy metals leads to the formation of toxic sludge, while on the other hand, membrane filtration accompanies fouling and regular maintenance. These limitations are known and have led to an extensive search for sustainable alternatives for lead remediation and to cut down the underlying cost associated with the process. This holds well for alternatives being developed for deployment in resource-limited settings like rural India (Malla et al., 2018).

In search of alternatives for conventional heavy metal remediation strategies, bioremediation has recently garnered significant attention. Biosorption refers to the passive binding of heavy metals to biological materials (Xie, 2024). Mechanisms involved in Biosorption include various physicochemical interactions such as ion exchange, complexation, adsorption, and precipitation. These interactions happen due to the presence of functional groups on the surface of biomass, which include carboxyl, hydroxyl, amino, and phosphate groups. These groups and their composition are inherent to the microorganism from which the biomass is generated. Microorganisms such as algae, bacteria, and fungi, along with various agricultural wastes, have been shown to exhibit heavy metal sequestration capabilities from contaminated wastewater (Göksungur et al., 2005; Awasthi et al., 2017). Yeast, among various microorganisms, has been acknowledged as a particularly promising candidate for an eco-friendly biosorbent. Yeast is abundant, grows rapidly, and has a cell wall rich with functional heavy metal binding moieties (such as carboxyl, amino, and phosphate groups) (Mohiuddin et al., 2024). Yeast biomass can also be acquired easily as a byproduct from breweries, food, and chemical industries, which makes it economically viable for large-scale applications (Bahafid et al., 2017).

Although individual yeast strains can effectively adsorb heavy metals, utilizing the consortium of yeast (a mixture of different yeast species working together) for bioremediation has attracted recent scientific interest, with several studies highlighting their advantages against single yeast strains (Kurade et al., 2017). The studies attributed the selection of consortium over single strains to the idea that synergistic interaction of yeast species can lead to better biosorption efficiency, stress tolerance in the presence of pollutants, and broader remediation possibilities. It’s possible that different yeast species co-existing in a polluted environment may show complementary metal binding abilities or may secrete extracellular polymers that can improve the metal capture efficiency. The resistance of a consortium towards environmental changes can be advantageous to real-world applications. Despite these ideas and hypotheses, the actual application of yeast consortium, especially in the context of immobilized systems, is still scant and underexplored (Mukherjee et al., 2021; Xu and Yu, 2021).

When considering immobilized systems, hydrogels are of quite an interest among the scientific community. Hydrogels are 3-D constructs of hydrophilic polymers that can retain a large amount of water while maintaining their structural integrity (Pickering et al., 2024). Hydrogels are highly porous, biocompatible, and have tunable physical properties, and hence are ideal for immobilizing living cells, including yeast. Immobilization of living cells could lead to benefits such as no loss of biomass during operation, recycling possibilities, and protection against environmental stress. The tunable nature of hydrogels allows the application of engineering principles that can lead to the optimization of mass transfer, mechanical strength, and stability. These properties could be exploited to use the hydrogen-encapsulated yeast cells for large-scale bioreactor applications (Albers et al., 2018; Castro-Gutierrez et al., 2022).

Hence, the integration of a yeast consortium into a hydrogel setup can be a novel and promising strategy for the development of advanced biosorbent materials. In this manuscript, we aim to demonstrate that the uniqueness of microbial biomass composition, combined with a systematic modelling approach, can be exploited to maximize metal removal. We aim to achieve the best possible remediation in the field by combining computational modelling and smart experiment designs. These optimizations we then want to bring over into the form of a bioaugmented filter to maximize lead removal from wastewater. We also want to move away from conventional bead encapsulation and move towards a gel slab setup, which can be combined easily with a conventional filter as an additional layer instead of relying upon the tight packing of beads. We aim to demonstrate that the immobilization could lead to the formation of an efficient bioaugmented filter system with potential for scaling up and industrial applications. We also aim to address several questions, viz., the optimal composition of native yeast consortium, the behavior of native yeast strains across varying environmental conditions, mechanism of bioremediation for our bioaugmented filter setup. We believe the learning from this work can be built upon in future studies to develop and scale up the performance of the filter for industrial applications.

## 2 Materials and methods

### 2.1 Optimization of Pb^++^ ions using selected strains

The optimization of lead removal for all three microbial strains was conducted using Design-Expert 13.0 software, utilizing statistical experimental designs to systematically assess and enhance biosorption efficiency. Key experimental variables, including pH, biomass dosage, and initial metal ion concentration, were selected based on preliminary studies and relevant literature. For each strain, a central composite design (CCD) was employed to evaluate the individual and interactive effects of these variables, enabling the development of predictive models for lead biosorption (Arakaki et al., 2011; Mishra and Malik, 2014). All experiments were performed in triplicate for each strain to ensure reproducibility, with appropriate blanks and controls included in every batch to account for non-biological removal and background interference (Rajpal et al., 2021). The collected data were analyzed using response surface methodology (RSM) within the software to determine the optimal conditions for maximum lead removal for each strain, and model adequacy was verified through analysis of variance (ANOVA) and diagnostic tools provided by the software.

### 2.2 Compatibility test

To assess the compatibility of microbial strains for consortium preparation, each isolate was first purified and tested for mutual compatibility by cross-streaking or co-culturing on YPD agar plates at 30 °C. Antagonistic interactions, such as inhibition zones or growth suppression, were monitored to identify incompatible strains (Lin et al., 2011). Only isolates exhibiting no antagonism were selected and combined into various consortium formulations. These formulations were then inoculated onto fresh YPD medium and incubated at 28 °C for 24 h to confirm co-existence, ensuring the final consortium comprised harmoniously interacting strains (Kannan et al., 2021).

### 2.3 Bioformulation and generating biomass

*Candida Tropicalis, Pichia kudriavzevii*, and *Clavispora lusitaniae* strains were grown separately in YPD broth for 24 h at 28 °C with agitation of 150 rpm until they reached the desired 1 OD. We then mixed the strains in an equal ratio to create the different combinations of consortium and adjusted the total volume to the desired level. We then transferred the mixed culture to a suitable medium and incubated it under optimal conditions. Once the consortium was stable and performing well, we used the consortium for further studies.

### 2.4 Biosorption of lead using consortia

After preparing the different combinations of consortium, the biomass was harvested by centrifugation at 4500 rpm for 15 min. The resulting cell pellets were washed twice with double-distilled water (ddH2O) to remove any residual media components and unbound ions, ensuring the purity of the biosorbent material. Batch biosorption experiments were conducted in 100 mL conical flasks containing aqueous solutions of lead and Cadmium at varying initial concentrations (e.g., 100, 150, 200 ppm and 50, 100, and 150 ppm), prepared from analytical-grade Pb (NO_3_)_2_ salts and Cd (NO_3_).4H_2_O. To each flask, 0.5 g of wet consortium biomass was added. Control flasks containing metal solutions without biomass were included to account for abiotic losses. The flasks were incubated at 25 °C for 24 h under static or shaking conditions, as required. After incubation, the mixtures were centrifuged at 4500 rpm for 10 min to separate the biomass from the solution. The supernatants were collected and analyzed for residual concentrations of Pb2 + using atomic absorption spectroscopy (AAS). The percentage removal of each metal was calculated by comparing the initial and final concentrations of each metal.

The Efficiency of biosorption [E (%)] was evaluated using a formula calculated by Rehman and Anjum, (2011); Aibeche et al. (2022):


(1) $$E\%=\left(\mathrm{Ci}-\frac{\mathrm{Cf}}{\mathrm{Ci}}\right)\times 100$$

Where Ci and *Cf* are determined as initial and final concentrations of individual metals, respectively (Units in mg/L).

### 2.5 SEM–EDX for consortium and bioaugmented filter

The Characterization of the surfaces of the Consortium and Bioaugmented filter before and after interaction with Cd and Pb was studied using SEM. For Consortium SEM analysis, the prepared metal-loaded and control consortium were fixed with 2.5% (v/v) glutaraldehyde at 4 °C overnight. The fixed sample was then applied and spread on the coverslip, followed by air-drying. And for Bioagumented filter, before proceeding for SEM- imaging along with EDX elemental analysis (C, N, O, Na, Ca, Pb), samples were dipped in liquid N_2_ to maintain the porosity of the Hydrogel slab. Yeast bioaugmented filter was fixed with the help of 2.5% (v/v) glutaraldehyde (0.1 M PBS, pH 7.4, 4 °C, 1 h) (Liaquat et al., 2020). Following fixation filter was washed with increased ethanol % (30 to 100% v/v, 10 min/step), then filter was washed with PBS buffer (0.1 M PBS, pH 7.4, 4 °C, 1 h) to remove remain fixative, after washing filter was kept for dehydration in hot air oven at 40 °C for 48 h and further sputter-coated with gold. These coated samples were investigated using a SEM instrument (CARL ZEISS).

### 2.6 Fourier transform infrared spectroscopy (FTIR) analysis for consortium

The following protocol was followed for preparing the sample for FTIR analysis. The biomass of the yeast consortium was collected after the biosorption study by centrifuging at 5000 rpm for 10 min to collect the pellet. For FTIR analysis, we have chosen the 100 mg/l concentration for both metals, lead and cadmium, and the control, yeast consortium, was taken without exposure to heavy metal concentration. The dried biomass was subsequently analyzed using a Fourier-transform infrared (FTIR) spectrometer, with spectra recorded in the 4,000–500 cm^−1^ range. This procedure enabled the assessment of biochemical changes in the yeast consortium in response to heavy metal exposure (Deokar et al., 2013).

### 2.7 Alginate yeast gel slab preparation

We cultivated the yeast consortium biomass to high cell density in a nutrient-rich YPD medium. The harvested biomass was then mixed with a 2% w/v aqueous sodium alginate solution. This mix was poured into a 47 mm diameter mold and layered with CaCl_2_ solution-soaked filter papers (0.45 mm) at its base. The hydrogel was further topped with a similar CaCl_2_ solution-soaked filter paper for proper crosslinking. The setup was finally left in a humid atmosphere at room temperature for 12 h or until the sandwiched consortium mix alginate solution had solidified into a hydrogel slab or bioaugmented filter.

### 2.8 Removal of lead by using bio-augmented filter

For the study to check the removal efficiency of lead by using a Bioaugmented Filter, the following procedure was followed. The bioaugmented filter or hydrogel slab was securely placed into the Vacuum Filtration Assembly unit. Lead solutions at concentrations ranging from 100 to 500 mg/L were prepared by diluting a stock Pb (NO₃)₂ solution, and each solution was passed through the filtration unit at a controlled flow rate, and the filtered solutions were collected in acid-washed flasks. For each concentration, the experiment was performed in triplicate, and a control filter prepared identically but lacking the Consortium biomass was included for comparison. The filtered solutions were analyzed for residual lead concentrations using atomic absorption spectrometry (AAS), ensuring accurate quantification. Removal efficiency was calculated by Equation 1, and statistical analysis was performed to evaluate the significance of differences between test and control filters.

### 2.9 Statistical analysis

The data collected in this study were statistically analyzed using JMP Pro 17 software. Mean values were assessed through a two-way analysis of variance (ANOVA), and the Tukey test was employed to determine the statistical significance of the results (*p* < 0.05). All results are expressed as the mean ± SE.

## 3 Results

### 3.1 Compatibility test

**Figure 1:**
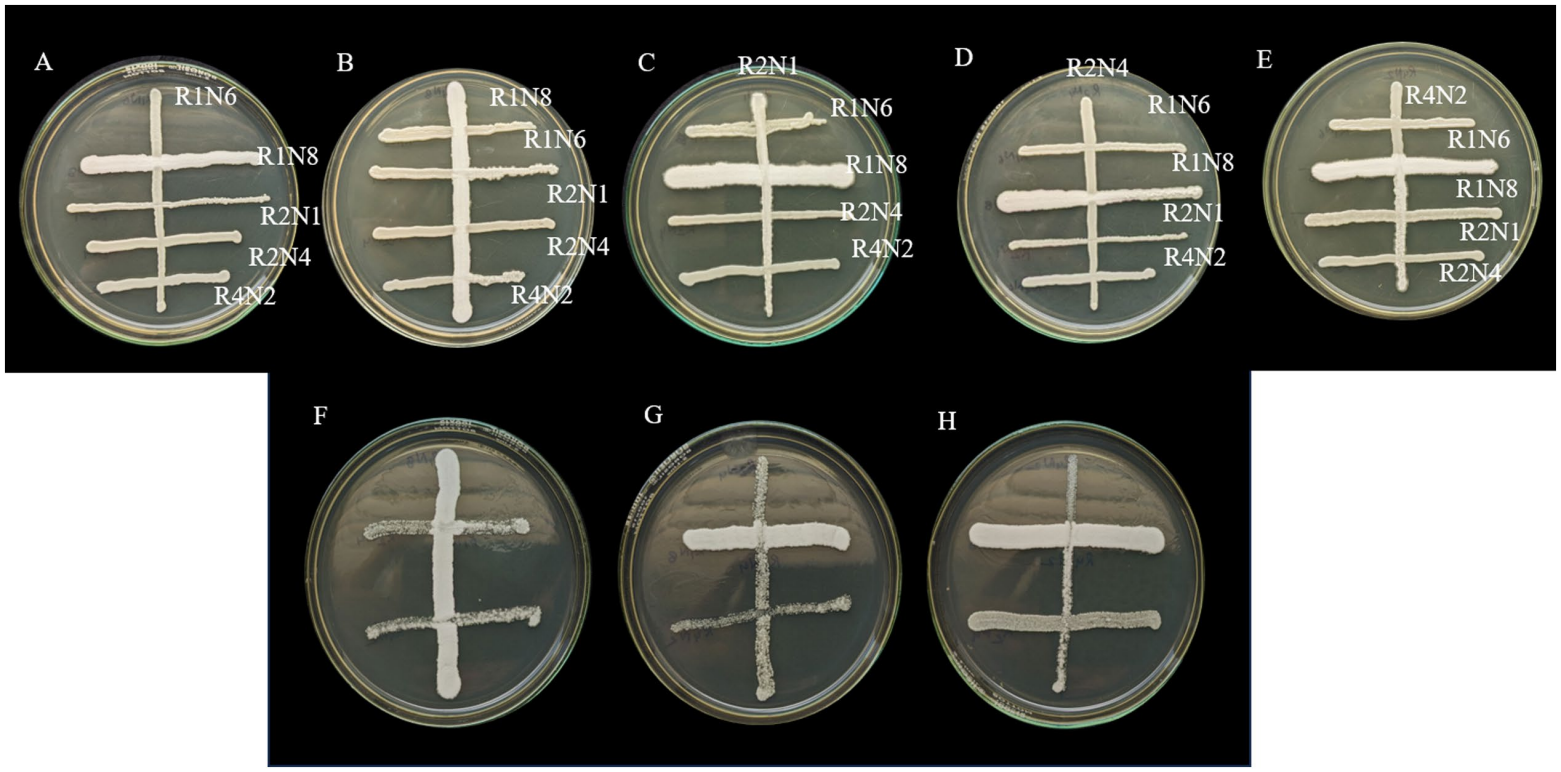
Compatibility testing of selected yeast strains. In Petri dishes labelled **A–E**, five strains (R1N6, R1N8, R2N1, R2N4, and R4N2) were cross-streaked to evaluate growth interactions. Plates **F–H** show similar assays among three strains (R1N8, R2N4, and R4N2). Distinct orientations and streak densities reveal varied microbial responses and potential consortium compatibility.

The compatibility test was performed to verify whether the top 5 chosen strains could co-exist or not. These five strains were later identified via Sanger sequencing and were identified as *Pichia kudriavzevii, Clavispora lusitaniae,* and three strains as *Candida Tropicalis*. Of the five, three strains, namely R1N8, R2N4, and R4N2 (*Pichia kudriavzevii, Candida Tropicalis,* and *Clavispora lusitaniae*), were chosen based on their excellent heavy metal (Cd and Pb in focus for this study) tolerance and remediation capabilities. All five strain combinations and three strain ones were found compatible with each other. These results encouraged the further development of consortia using these strains for the remediation of Pb and Cd and filter augmentation studies (Figure 1).

### 3.2 Establishment of response surface methodology

We used Response Surface Methodology (RSM) to generate models to optimize parameters for bioremediation for individual yeast strains. Box–Behnken design (BBD) matrix was employed for the modelling in Design Expert software (Version 13.0). Three key variables were modelled, viz. pH, Biomass, and concentration of Pb^2+^ (Supplementary Table S1). The combinations of experiments (Table 1) were performed following the guidance from the software, and the corresponding lead removal data were fed back, which resulted in the model.

**Table 1:** Experimental and predicted response for Pb^++^ ions biosorption using *Pichia kudriavzevii, Candida tropicalis,* and *Clavispora lusitaniae.*

No. of experiments	Pattern	pH	Biomass (g)	Pb Concentration (mg/L)	Removal % (*Pichia kudriavzevii*)		Removal % (*Candida tropicalis*)		Removal % (*Clavispora lusitaniae*)	
		A	B	C	Experiment value	Predicted value	Experiment value	Predicted value	Experiment value	Predicted value
1	+−−	7	0.5	100	57.3	52.6	97.7	98	94.8	95.7
2	00a	5	1.25	100	99.9	98.1	99.9	96.9	97.9	98.7
3	++−	7	2	100	99.4	100.4	99.9	98	99.6	96.5
4	+ − +	7	0.5	200	38.9	31.6	77.8	72.4	60.1	57.8
5	0	5	1.25	150	99.8	84.7	98.5	94.5	99.6	97
6	a00	3	1.25	150	38.8	31.5	68.7	63.6	62.7	60.4
7	+++	7	2	200	94.8	94.7	99.7	99	99	98.6
8	00A	5	1.25	200	97.8	103.5	91.5	97.2	94.9	96.6
9	0A0	5	2	150	82.2	71.3	99.8	98.7	99.8	103.2
10	−−−	3	0.5	100	1.9	1	10.8	10.8	9.1	8.8
11	0a0	5	0.5	150	21.5	36.3	50.5	54.3	55.9	55.2
12	A00	7	1.25	150	73.6	84.7	99.5	107.2	99.3	104.2
13	−++	3	2	200	36	39.8	99.9	99	99.4	97.8
14	− + −	3	2	100	1.7	8	68.2	72.9	62.5	64.2
15	−−+	3	0.5	200	19.6	17.6	9	10.2	0	2.5
16	0	5	1.25	150	77.4	84.7	95.9	94.5	99.6	97

#### 3.2.1 Experimental and predicted response for Pb^2+^ biosorption using *Pichia kudriavzevii*

pH, Pb^2+^ concentration, and biomass dosage were modelled within the physiological ranges suitable for our applications, i.e., pH: 3–7, Pb concentration: 100–200 mg/L, and Biomass: 0.5–2 g. The regression model generated for *Pichia kudriavzevii* showed a strong positive effect of both pH and biomass on the bioremediation efficiency. On the other hand, higher initial Pb^2+^ concentrations lead to reduced bioremediation efficiency. A synergistic effect was observed between pH and biomass, which enhances removal when both are increased. While interactions involving Pb^2+^ concentration and higher-order (squared coefficients) terms were found to be negative. This suggests that excessive levels of these combinations resulted in reduced biosorption, which could be due to the saturation of metal binding sites.

Biosorption (%) of Pb2 + using *Pichia kudriavzevii*


$$\begin{array}{l} R=-135.875+85.4269*A+111.556*B+-1.52759*C+\\ \;6.81604*\mathrm{AB}+-0.0937969*\mathrm{AC}+0.101292*\mathrm{BC}+\\ \;-6.65785*A2+-54.998*B2+0.00641411*C2 \end{array}$$

A–coefficients for pH.

B–coefficient for biomass concentration.

C–coefficient for the initial Pb^2+^ concentration.

AB–coefficient of the interaction term between pH and biomass.

AC and BC – coefficient of interaction terms involving Pb^2+^ concentration.

**Figure 2:**
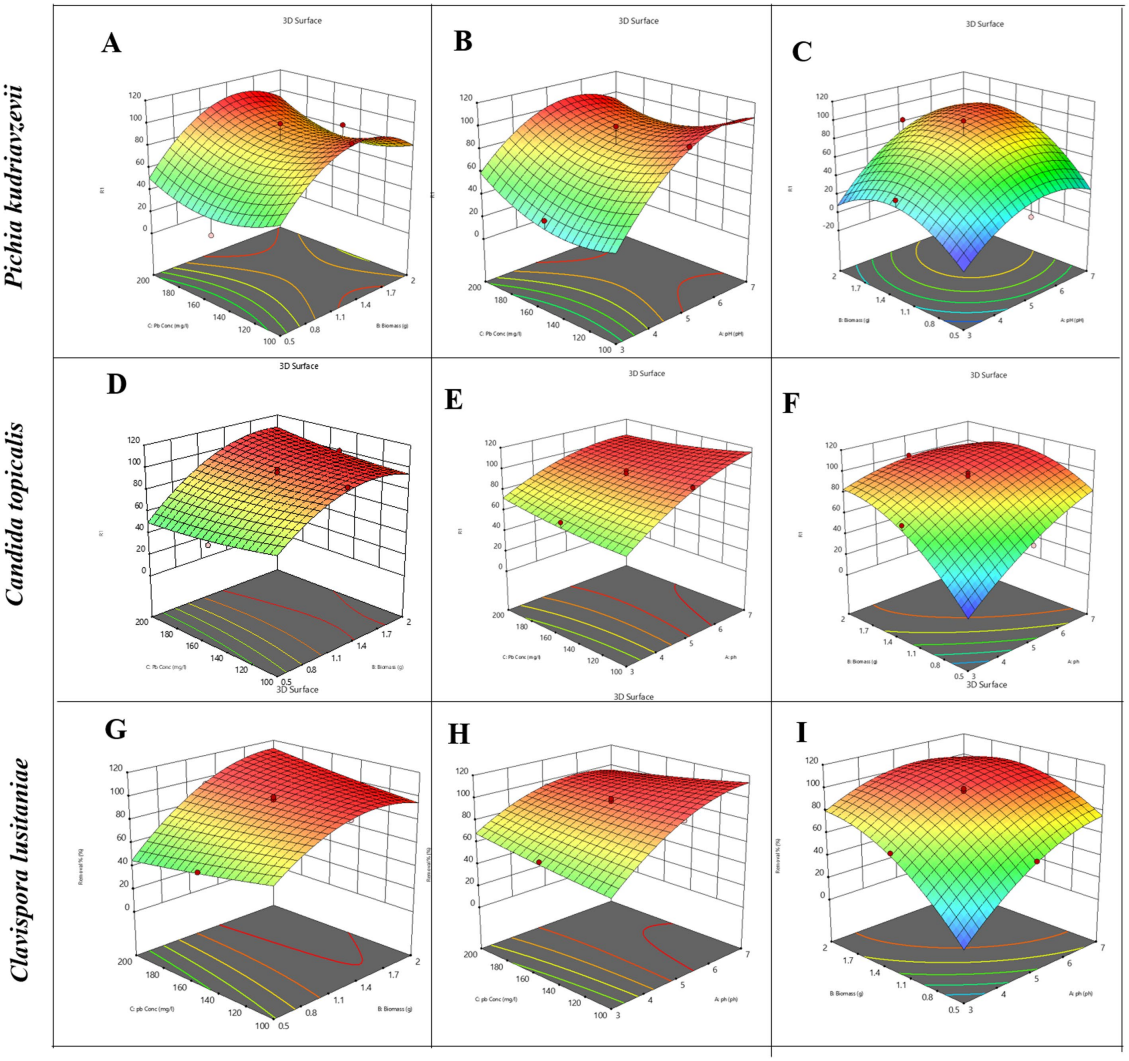
A grid of nine 3D response surface plots demonstrates the combined effects of pH, biomass (g), and lead concentration (Pb, mg/L) on removal efficiency (%) for three yeast species: Pichia kudriavzevii **(A–C)**, Candida tropicalis **(D–F)**, and Clavispora lusitaniae **(G–I)**. Each plot visualizes the response surface with a color gradient ranging from red (highest removal) to blue (lowest removal), with contour lines mapping removal variation. The axes in each plot represent pairwise combinations of the independent variables, specifically: biomass versus Pb concentration **(A,D,G)**; pH versus Pb concentration **(B,E,H)**; and pH versus biomass **(C,F,I)**.

The value of the coefficients in the equation above indicates the level of impact the corresponding factors have on the removal of lead. We observed that for this strain, pH and biomass were significantly important factors than Pb^2+^ concentration for heavy metal removal. The analysis of variance (Supplementary Table S2) for the generated equation showed that the model is highly significant (*F*-value = 12.90, *p* < 0.05) and fits well. Lack of fit (*F* = 0.57) was found to be not significant, and the model was able to predict with high accuracy (adjusted *R*^2^ = 0.8772, predicted *R*^2^ = 0.6845, with a difference less than 0.2). The cube plot (Supplementary Figure S2), 2D plot (Supplementary Figure S1), 3D plot (Figure 2), and response surface analysis suggest that maximum biosorption of Pb^2+^ was achieved at pH 7, higher biomass dosage at 2 g, and lower initial Pb^2+^ concentration of 100 mg/L for this yeast strain. These conditions were designated optimal for maximal removal of lead. Overall, we learned from the model that both pH and biomass can be optimized to gain efficiency while keeping the Pb^2+^ concentration moderate for strain *Pichia kudriavzevii.*

#### 3.2.2 Experimental and predicted response for Pb^2+^ biosorption using *Candida tropicalis*

A similar set of experiments was performed for the other strain, i.e., *Candida tropicalis.* The ranges for variation of all three parameters were kept the same. In this case, the regression analysis showed that the pH and biomass again had a positive effect on lead removal, while on the other hand, Pb^2+^ concentration had a minor but positive effect. Significant negative coefficients for square terms revealed that excessively high values of individual variables will impact the metal removal negatively. The coefficient for the combination of biomass and lead concentration has a positive effect on metal removal, while on the other hand, the combinations of pH and biomass and pH and concentration can reduce the metal removal for certain combinations.

Biosorption (%) of Pb2 + using *Candida tropicalis*


$$\begin{array}{l} R=94.47+21.8077*A+22.1723*B+0.1275*C+-15.5287\\ \;*\mathrm{AB}+-6.2425*\mathrm{AC}+6.65875*\mathrm{BC}+-9.05333*A2+\\ \;-17.9683*B2+2.59583*C2 \end{array}$$

The model obtained predicted a maximum lead biosorption capacity for *Candida Tropicalis* of 94.5% under the best conditions. The analysis of variance (Supplementary Table S3) for the generated equation showed that the model is highly significant (*F*-value = 43.21, *p* < 0.05) and fits well. Lack of fit (*F* = 12.97) was suggested to be not significant, and the model was able to predict with high accuracy (adjusted *R*^2^ = 0.9620, predicted *R*^2^ = 0.8566). The cube plot (Supplementary Figure S3), 2D plot (Supplementary Figure S1), 3D plot (Figure 2), and response surface analysis suggest that maximum biosorption of Pb^2+^ was achieved at pH 7, higher biomass dosage at 2 g, and higher initial Pb^2+^ concentration of 200 mg/L for this yeast strain. These conditions (highest levels for all three factors) were designated optimal for maximal removal of lead, i.e., up to 99% for *Candida Tropicalis*.

#### 3.2.3 Experimental and predicted response for Pb^2+^ biosorption using *Clavispora lusitaniae*

For the strain *Clavispora lusitaniae*, the previous set of experiments and regression analysis indicated strong positive linear effects of pH (+21.71) and biomass (+23.83) on lead removal. On the other hand, Pb^2+^ concentration had a negative effect (−0.85). Significant negative coefficients for square terms revealed that excessively high values of individual variables will impact the metal removal negatively. The negative coefficient for combinations of pH/biomass and pH/ Pb^2+^ concentration showed that certain combinations of these parameters can reduce metal removal efficiency. On the other hand, a certain combination of biomass and lead concentration could have a positive impact on metal removal.

Biosorption (%) of Pb2 + using *Clavispora lusitaniae*


$$\begin{array}{l} R=96.442+21.7059*A+23.8338*B+-0.8525*C+\\ \;-13.3903*\mathrm{AB}+-8.13344*\mathrm{AC}+9.74844*\mathrm{BC}+\\ \;-14.3796*A^{2}+-17.5046*B^{2}+0.994943*C^{2} \end{array}$$

The model obtained predicted a maximum lead biosorption capacity for *Clavispora lusitaniae* as 96.4% under the best conditions. The analysis of variance (Supplementary Table S3) for the generated equation showed that the model is highly significant (*F*-value = 152.25, *p* < 0.05) and fits well. Lack of fit (*F* = 6.29) was suggested to be not significant, and the model was able to predict with high accuracy (adjusted *R*^2^ = 0.9891, predicted *R*^2^ = 0.9568). The cube plot (Supplementary Figure S4), 2D plot (Supplementary Figure S1), 3D plot (Figure 2) and response surface analysis suggest that the metal removal efficiency increases significantly at pH 5.5 to 7 and biomass dose above 1.4 grams. The metal removal is almost 100% at moderate to high Pb^2+^ concentrations for *Clavispora lusitaniae*. This level of removal sometimes exceeding a bit more than 100% suggests that beyond surface adsorption, other phenomena such as intracellular accumulation or precipitation might be involved. In this scenario, the model was able to predict strongly the effect observed in the cube plot (Supplementary Figure S4), i.e., the systematic progression of metal removal.

Overall, we found that all three strains have excellent metal removal properties at a pH of around 7 and higher biomass dosage. All the predicted regression models seem to be quite reliable in predicting the metal removal for our strains. These conditions provide us with a solid foundation for further consortium design, design of filter experiments, and further practical application in the future.

### 3.3 Biosorption of Cd and Pb by using consortia

**Figure 3:**
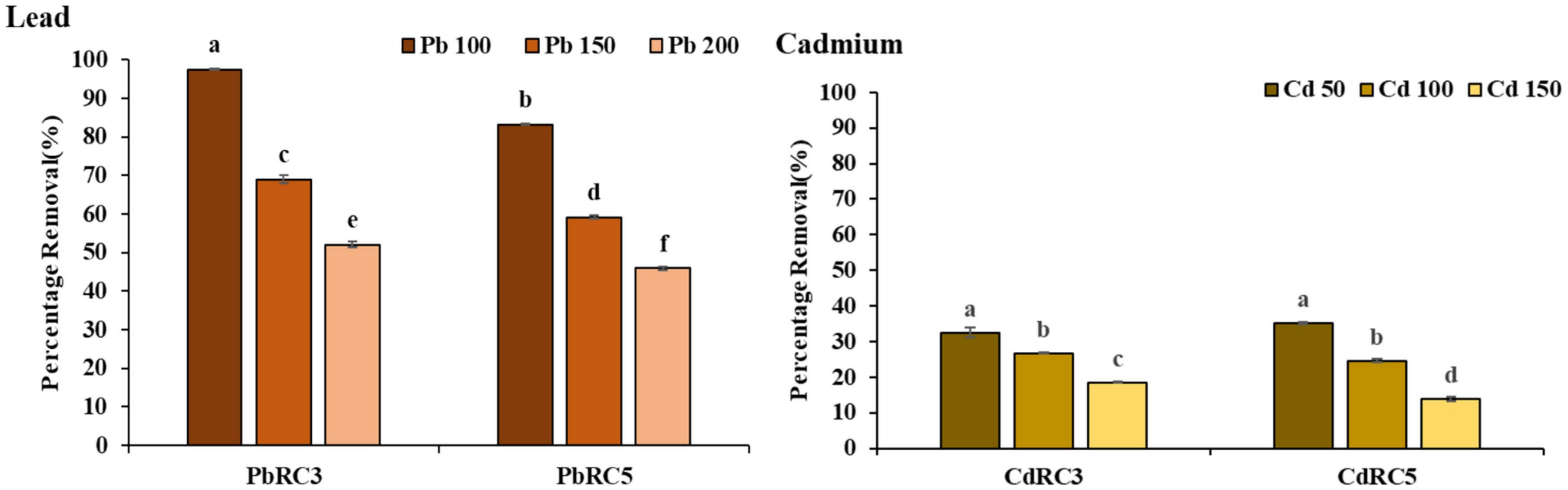
Biosorption of Lead and Cadmium by using two different combinations of the consortium at Different concentrations. Vertical bars indicate Mean ± SE. Means denoted by a small letter represent Means denoted by a different letter are significantly different at *p* ≤ 0.05 according to Tukey’s studentized range test.

We further evaluated the bioremediation capacity (Figure 3) of two initial consortia, namely PbRC3 (mix of three strains in equal proportions) and PbRC5 (mix of five strains in equal proportions), over a gradient of lead concentration (100–200 mg/L). We found that the PbRC3 consortium showed a maximum lead removal efficiency of 97.49% at an initial lead concentration of 100 mg/L, which declined further with increasing initial lead concentrations. This shows a negative correlation between lead removal to initial heavy metal concentrations. On the other hand, the PbRC5 consortium achieved a removal efficiency of 83.22% at 100 mg/L of initial lead concentrations, which also declined with further increase of initial lead concentrations. This result showed that the 3-mix consortium seems to outperform the 5-mix one consistently over a wide range of initial lead concentrations.

A similar set of experiments was also conducted for cadmium (Figure 3) with 3-mix and 5-mix consortia. The results followed a similar trend of decrease in metal removal with the successive increase of initial cadmium concentrations. Although the 5-mix consortium (35.28% at 50 ppm) here had a higher metal removal when compared to the 3-mix consortium (32.53% at 50 ppm), overall metal removal was never >36%. Hence, we focused on lead removal from here on out.

**Figure 4:**
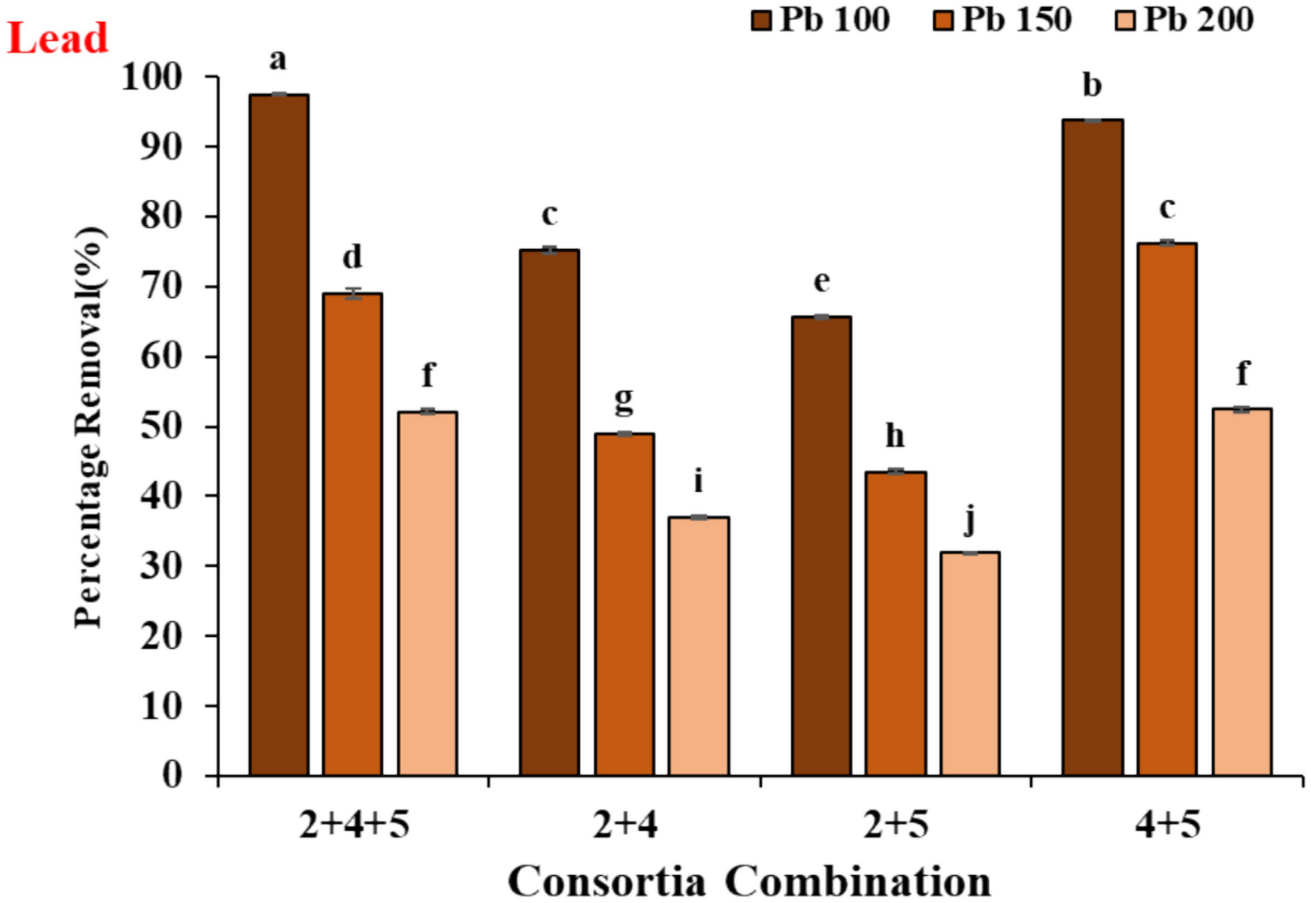
The lead (Pb) removal efficiency of various yeast consortium combinations (2 + 4 + 5, 2 + 4, 2 + 5, and 4 + 5) was assessed at different lead concentrations (Pb 100, Pb 150, and Pb 200). Vertical bars indicate Mean ± SE. Means denoted by a small letter represent Means denoted by a different letter are significantly different at *p* ≤ 0.05 according to Tukey’s studentized range test.

For further optimization of lead removal with the 3-mix consortium and understanding which combinations of two out of three work best, we repeated the metal removal experiments (Figure 4).

The experiments allowed us to realize that the combination of 4th and 5th strains out of the 3-mix consortium (mixture of 2nd, 4th, and 5th strains) performed on par with the 3-mix consortium and better than the rest of the 2-mix consortia. Our findings suggest maximum contribution of strains 4 and 5 towards lead removal in the designed consortia, while we think strain two might have some underlying synergistic effects stabilizing the consortia.

### 3.4 SEM analysis for the consortium

**Figure 5:**
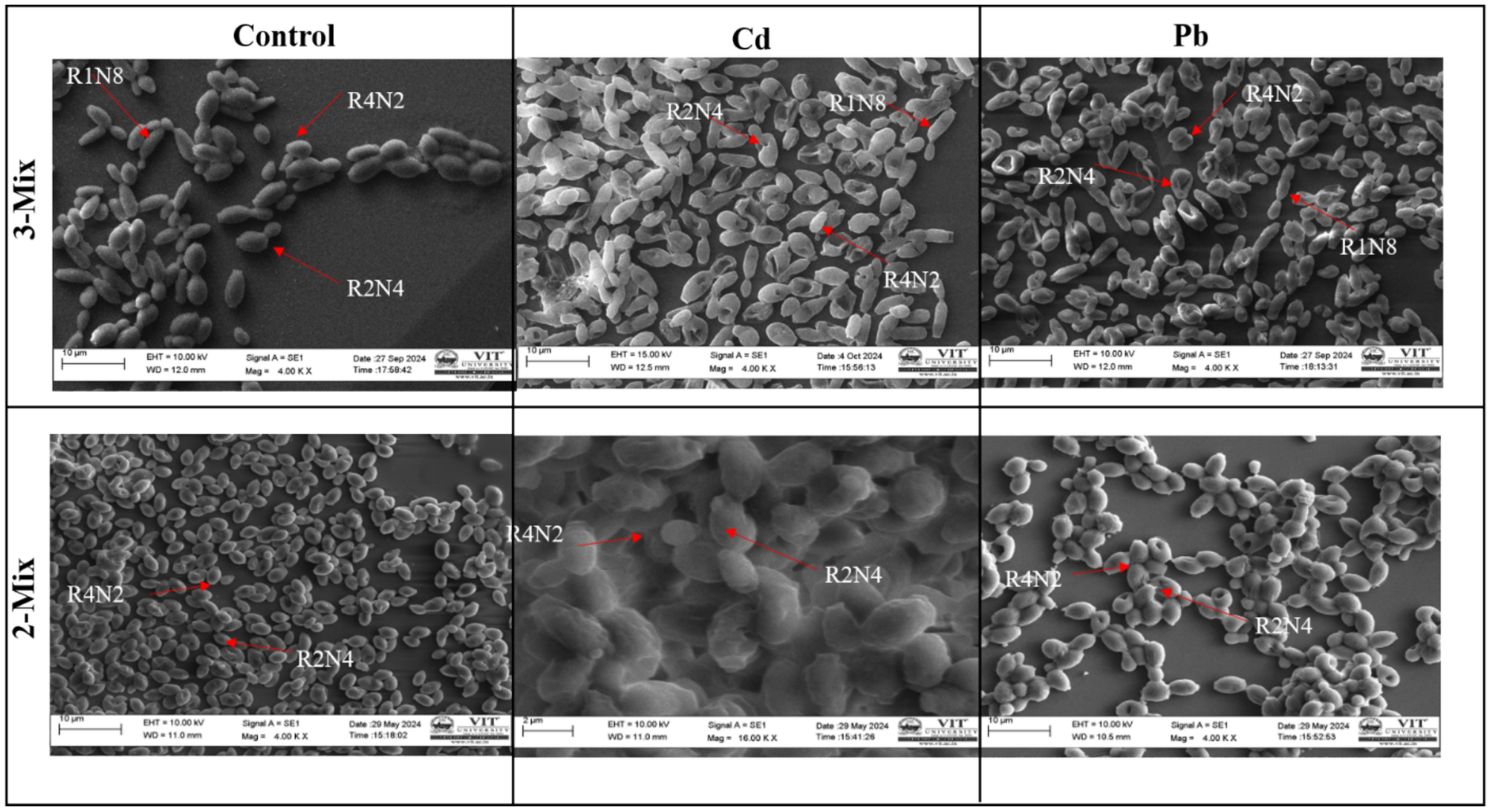
SEM analysis for the consortium.

SEM imaging allowed us to visually confirm (Figure 5) the process of heavy metal removal by the 2-mix and 3-mix strains, and the morphological variations between the control and metal-exposed consortium were evident. For the SEM imaging, lower end of lead and cadmium concentrations were used, i.e., 100 mg/L and 50 mg/L. The signs of heavy metal stress come in the form of increased surface roughness and irregularities.

### 3.5 FTIR analysis for consortia

**Figure 6:**
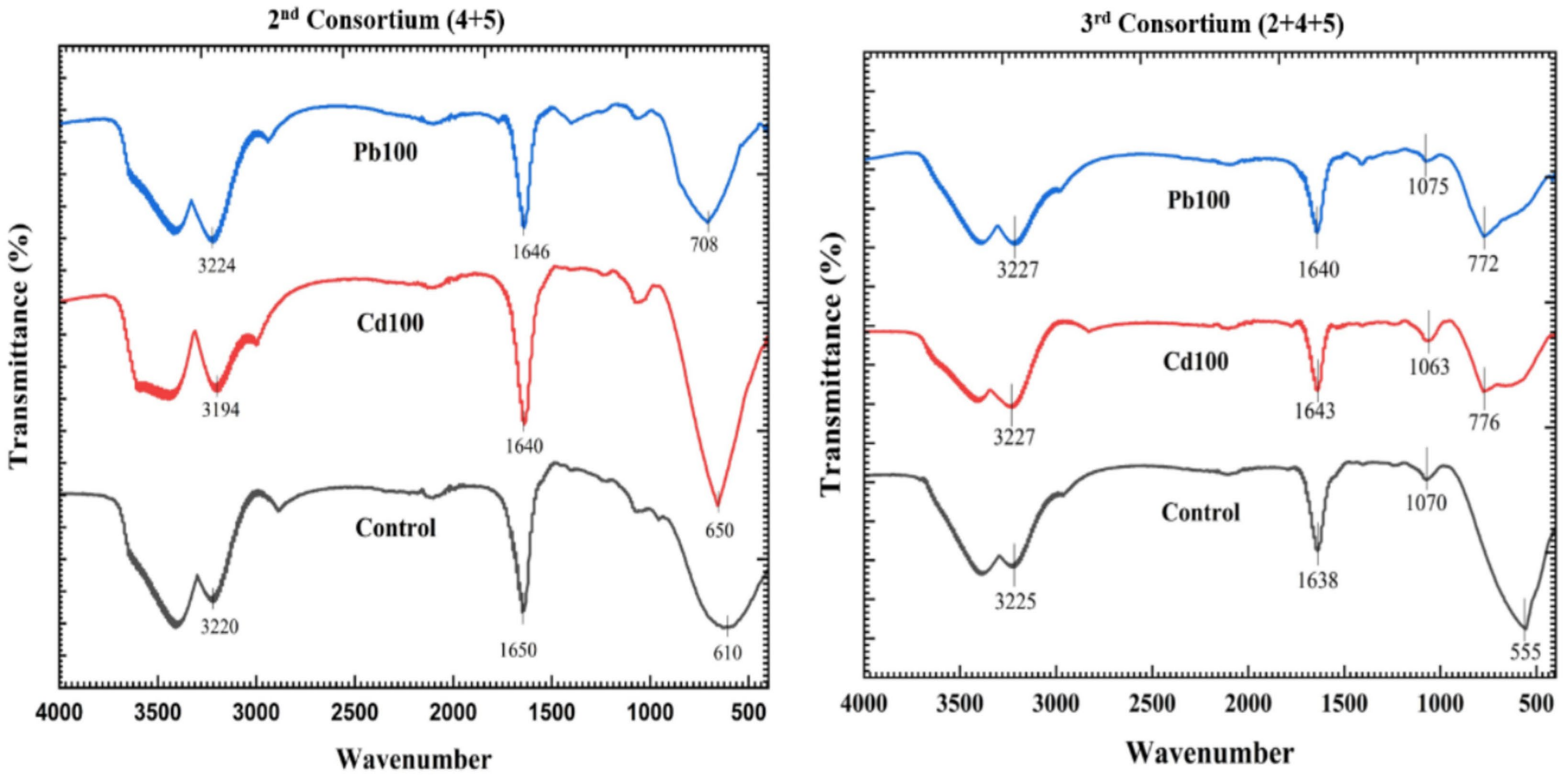
FTIR analysis for the consortium.

Spectra for the 2-mix (4 + 5) and 3-Mix (2 + 4 + 5) consortia at lead and cadmium exposure of 100 ppm each revealed a distinct shift in peak at specific wave numbers (Figure 6). For the 2-mix consortium, the broad absorption bands around 3,224 cm^−1^ (for Pb100), 3,194 cm^−1^ (for Cd100), and 3,220 cm^−1^ (for control) correspond to O-H and N-H interactions. The region observed at 1646 cm^−1^ (for Pb100), 1,640 cm^−1^ (for Cd100), and 1,650 cm^−1^ (for control) seems to point towards possible carboxyl group interactions. Although these changes were minimal, we observed a distinct change in the signature region associated with metal-specific binding, i.e., 708 cm^−1^ (for Pb100), 650 cm^−1^ (for Cd100), and 610 cm^−1^ (for control).

For the 3-mix (2 + 4 + 5) consortium, the first two regions were found to be consistent and pointed towards no O-H and N-H interactions as well as no carboxyl group interactions. However, similar to the 2-mix consortium, there was a significant wave shift in the signature region associated with metal binding, i.e., 772 cm^−1^ (for Pb100), 776 cm^−1^ (for Cd100), and 555 cm^−1^ (for control). The shift in the case of the 3-mix consortium was much higher than the 2-mix consortia.

### 3.6 Generate biomass and alginate yeast gel slab preparation

We were successful in generating alginate gel slabs by soaking a confined amount of sodium alginate sandwiched between filter papers soaked with Calcium chloride (Supplementary Figure S5). This formed the basic filter and the base for all our experiments. For biomass generation, which was later used to supplement the filters. We grew yeast strains in liquid medium and then prepared different consortia, which were then pelleted down to achieve a constant biomass for different sets of experiments.

The gel slab format allowed the pseudo-metal solution to pass through while providing ample interaction with entrapped yeast cells. Our gel slabs had an average diameter of 47 ± 0.5 mm, thicknesses of 2.0 ± 0.1 mm, and a volume of 4 mL (Supplementary Table S5). We were able to consistently produce equivalent control and biomass-laden gel slabs.

### 3.7 Evaluating the effectiveness of bio-augmented filters for lead removal

**Figure 7:**
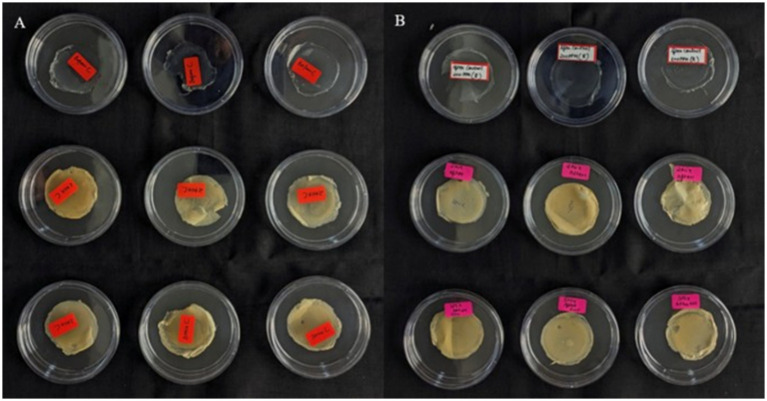
Visual comparison of bio-augmented filters before and after lead exposure.**(A)** Untreated filters: first row – control; second row – 2-mix; third row – 3-mix. **(B)** Filters after treatment: first row control; second row 2-mix; third row 3-mix.

**Figure 8:**
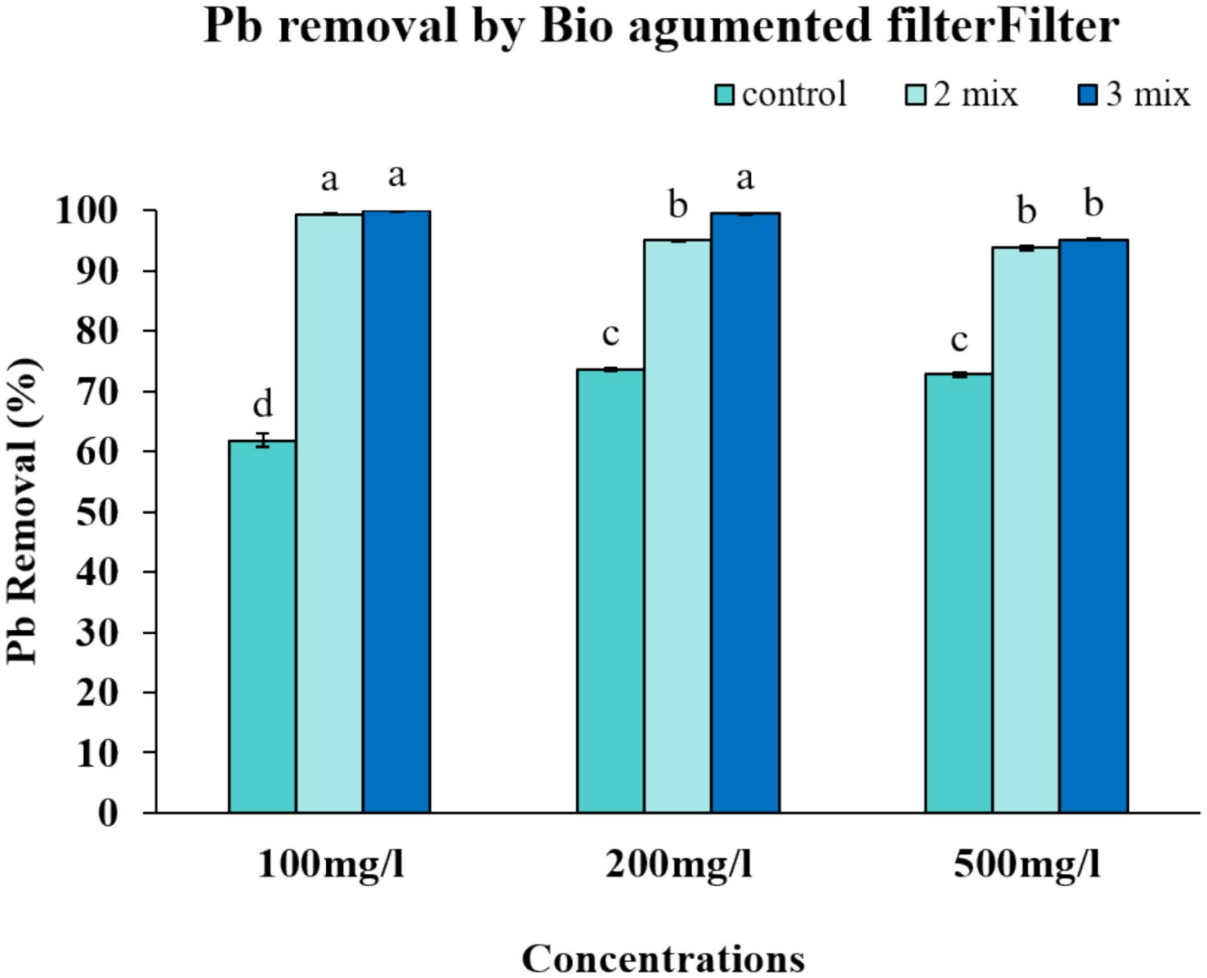
Lead removal by bio-augmented filter. Vertical bars indicate Mean ± SE. Means denoted by a small letter represent Means denoted by a different letter are significantly different at *p* ≤ 0.05 according to Tukey’s studentized range test.

We performed a series of experiments to evaluate the efficiency of our proposed filter design. We focused on assessing the filter’s efficiency in removing lead at different levels of lead in the solution (Figure 7). We observed that the 2-mix filter was able to achieve an impressive level of metal removal up to 99.39% while the 3-mix filter was able to achieve 99.97% metal removal at 100 ppm. The improved metal removal worked even for 500 mg/L lead solution, where 2-mix filter and 3-mix filter maintained the efficiency at 93.77 and 95.19% respectively, which was much better than the control or the consortium alone could achieve (Figure 8). The percentage removal was statistically significant. Our results demonstrate that our yeast bio-augmented filter is effective for lead removal up to 500 ppm.

### 3.8 SEM–EDX analysis for bioaugmented filter

Finally, we confirmed the removal of metal by control, 2-mix, and 3-mix filters visually with the help of scanning electron microscopy. The SEM–EDX data confirmed the qualitative removal of lead from the metal solution, as well as verifying the mode of metal removal being biosorption (Table 2).

**Table 2:** EDX elemental mapping of the yeast bioaugmented filter.

	Before	After
Control	0.03	0.36
2 Mix	0.00	0.38
3 Mix	0.00	0.24

The atomic% for each Filter Before and after treatment of Lead.

**Figure 9:**
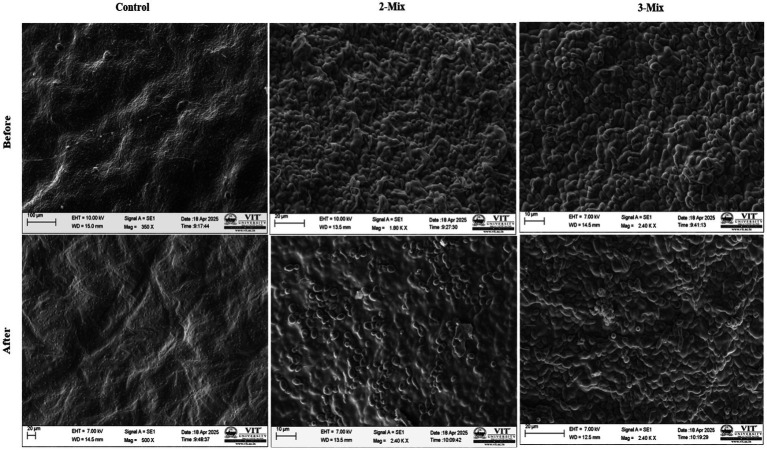
SEM analysis for the Bio-augmented filter before and after treatment.

**Figure 10:**
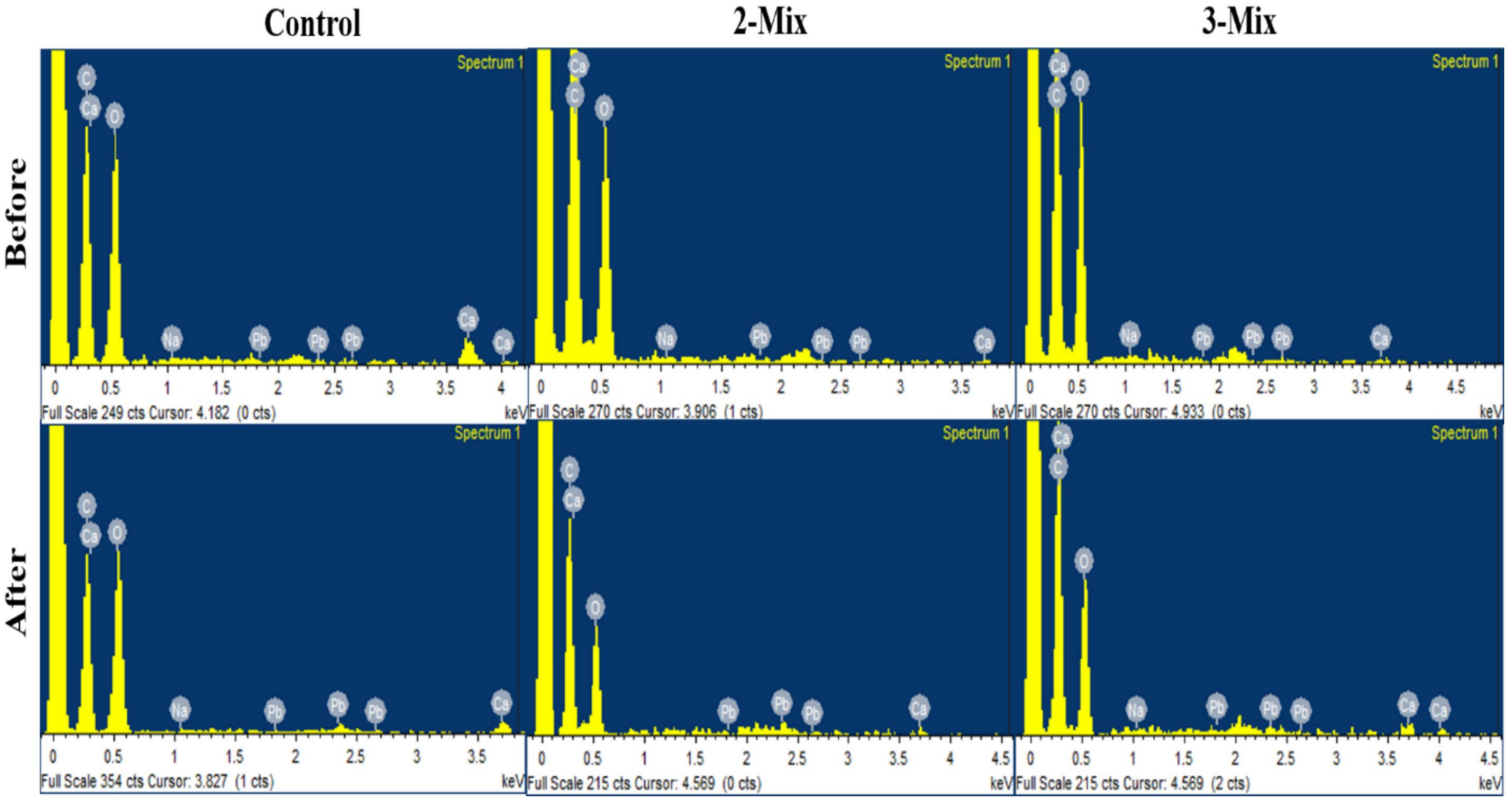
EDX analysis for the Bio-augmented filter before and after treatment.

In Figure 9, we can see the distinct differences between the control filter exposed to heavy metal and the bioaugmented 2-mix and 3-mix filters exposed to similar heavy metal exposure. The analysis of EDX data shows significant retention of heavy metals post heavy metal exposure in all cases. The heavy metal presence before heavy metal exposure is insignificant. This data is qualitative, not quantitative, because the presence of heavy metal detected depends on the section of the filter construct being examined, which differs among the types being compared. The morphological changes observed during SEM and all the experimental data validate the hypothesis of enhancement of heavy metal removal upon the incorporation of the consortium into the filter construct (Figure 10).

## 4 Discussion

Our study sheds light on the untapped potential of a native consortium made of yeast strains for the remediation of Lead. We were also successful in showcasing the additive effect of these consortia on lead removal from artificial polluted water samples when used in tandem with the conventional filters. Our initial compatibility studies on the three chosen strains, viz. *Pichia kudriavzevii* (R1N8), *Candida tropicalis* (R2N4), and *Clavispora lusitaniae* (R4N2) yielded exciting results, revealing the compatible nature of all three strains with each other. The strains grow well together and co-exist in YPD medium, making them excellent candidates for consortium development. Our experimental 2-strain (2-mix, R2N4 + R4N2) and 3-strain (all three) consortium worked well for the removal of lead in 100–200 ppm concentration ranges, demonstrating higher efficiency. The 2-strain consortium (2-mix) was able to remove 93.77% of lead at 100 ppm and 52.42% of lead at 200 ppm from artificial samples. The 3-strain consortium (3-mix) was able to remove 97.49% of lead at 100 ppm and 52.11% of lead at 200 ppm, which was at least on par or better than the 2-strain consortium. These results allowed us to state that the synergistic interactions enhance the biosorption of lead in the yeast consortium, particularly at lower heavy metal concentrations. This insight is further supported by the findings from other studies in this area on mixed microbial systems (Wang et al., 2021; Kumar et al., 2022; Zeng et al., 2022). The modelling of the metal removal of individual strains helped us significantly in the consortia design and optimization experiments. Especially the knowledge of conditions to achieve maximum metal removal efficiency allowed us to find the sweet spot where the metal removal by all component strains will be optimal and hence result in maximum remediation overall.

As far as the tandem application in conventional filters is concerned, our results demonstrate further improvement in the lead removal efficiency for these bio-augmented filters. The 2-strain mix, when applied to an alginate-based filter, demonstrates lead removal from artificial solutions up to 99.39% at 100 ppm and 93.77% at 500 ppm. For the 3-mix consortium, after addition to the alginate filter, it showed 99.97% removal at 100 ppm and 95.19% at 500 ppm. This level of efficiency (Table 3) surpasses multiple conventional biosorbents and emphasizes the importance of bioaugmentation, which holds particularly well for higher contaminant loads (Razzak et al., 2022; Ayach et al., 2024). Although we cannot verify exact physiological parameters inside the hydrogel, which is protected from the environment. The porosity of 2% alginate is still in the nanometer range, which allows free passage for the heavy metals to interact with the yeast biomass. We do expect that the effective exposed yeast surface when embedded in the alginate might be a bit less, but the factor should be a constant for the whole filter. Also, from our experiments, it’s clear that the incorporation of yeast has an additive effect in filter performance. The alginate alone can at max reach a removal efficiency of up to 75% for lead. Thus, the optimization of the batch experiments can still be relied upon for filter optimization to improve this additive effect of using yeast biomass. One can also design a set of experiments to model the filter performance exclusively. However, that is beyond the scope of this article.

**Table 3:** Removal efficiency of Heavy metals by different immobilized yeasts.

Sl. No.	Yeast encapsulated with alginate	Targeted metal	Removal efficiency	Operational details	References
1	*Candida krusei*	Cu (II)	0.687 mmol/g	3.6% Sodium alginate used to encapsulate	Luk et al. (2017)
2	*Saccharomyces cerevisiae*	Cu (II), Pb (II), Cd (II), and Zn (II)	Cu (85–91%), Pb (93–97%), Cd (80–85%), and Zn (70–75%)	Composite beads of brewing residual *S. cerevisiae* and sodium alginate, pH 5–6, and temperature 25 °C	De Rossi et al. (2020)
3	*Saccharomyces cerevisiae*	Cr (III)	98.5–98.6%	3% alginate beads	Mahmoud and Mohamed (2017)
4	*Penicillium janthinellum* GXCR	Cu, Pb, Cd	98.9, 95.5 and 84.8%	Conidia immobilized in 2% PVA-3% SA beads	Cai et al. (2016)
5	*Saccharomyces cerevisiae*	Zn, Cu	Aerobic, Ca-alginate: Zn: 99.76% Cu: 91.7%, Anaerobic, Ca-alginate: Zn: 0.8% less than aerobic, Cu: 2.1–9.9% less than aerobic	Tested under both aerobic and anaerobic conditions	Garanin and Lykov (2024)
6	*Pichia kudriavzevii YB5*	Cd (II)	54.35%	Immobilized with 2% (w/v) sodium alginate, 2% (w/v) calcium chloride, and polyurethane foam	Zhao et al. (2018)
7	*Saccharomyces cerevisiae*	U(VI)	142.1 μmol/g	mobilized in beads composed of 5% (w/v) PVA, 1% (w/v) alginate, 0.05% (w/v) graphene oxide (GO), 2% (w/v) active dry yeast	Chen et al. (2020)
8	*Rhodotorula mucilaginosa*	Se (VI)	43%	Alginate bead hydrogel immobilization	González-Morales et al. (2025)

Further mechanistic studies using SEM and FTIR support the observation, i.e., the additive effect and improved heavy metal removal efficiency. The SEM images also enabled us to observe the surface metal deposition on the yeast cells, part of the consortium, confirming the hypothesis. It was also evident that the heavy metal stress and deposition led to a change in morphology. The analysis of FTIR data showed peak shifts, which can be attributed to the changes in functional group profiles upon interaction with lead. This supports the idea of biosorption facilitated by the cell wall of yeasts, which is composed of polysaccharides and proteins. The much greater shift in 3 mix consortia compared to the 2 mix ones in terms of control and treated yeasts could be attributed to the fact that both *Candida tropicalis* (4) and *Pichia kudriavzevii* (2) form strong biofilms, while *Clavispora lusitaniae* (5) forms weak or moderate biofilms in the absence of heavy metals. Also, the biofilms are rich in polysaccharides, extracellular proteins, and lipids. This could explain that in 3-mix consortium (made up of 2 + 4 + 5) has higher biofilm presence and hence a higher shift in FTIR peak than the 2-mix consortium (made up of 4 + 5) in the absence of heavy metal. Other evidence from the qualitative SEM–EDX data revealed metal deposition both at the yeast cell and alginate filters. This validates the experimental data and supports our study.

Overall, we can state that both the consortium mixes positively augment the lead filtration capabilities of the bioaugmented filter and maintain relevance even at an elevated presence of the contaminant. This study has its limitations since it was conducted in a controlled laboratory setting, and the phenomenon of adsorption being driven by interaction kinetics might behave differently in a real-world setting, which may include interaction with nonspecific contaminants. This challenge can be eliminated via a smart filter assembly where our augmented filter could be a final layer among a series of conventional filters. Although the study was conducted in controlled laboratory settings, we are confident that the underlying physical and chemical processes involved in the filtration would not differ much in real-world applications. Our proposed filter can find usage at an industrial scale. Still, we agree collectively that further research and validation in that direction are still missing and should be undertaken shortly. We must assess the long-term stability, reusability, and temporal performance of the designed filter. Our study, with the help of a yeast consortium, offers a sustainable and scalable alternative to standard chemical-based heavy metal removal.

## 5 Conclusion

Our study demonstrates that it is possible to optimize the biosorptive capacity/performance of yeast strains via modelling. Optimization guided by computational modelling of experimental parameters such as pH, biomass, and concentration can significantly reduce the total number of experiments performed for these studies. Different strains can have different optimal conditions for efficient bioremediation. *Clavispora lusitaniae* and *Candida tropicalis* showed significant improvement in removal efficiencies following the optimizations. All three of our cultures were compatible, and to our advantage, were found to have a synergistic effect towards lead removal. Augmenting alginate filters with the biomass from the consortium leads to an increase in the filtration efficiency by reaching values such as 99.97% lead removal at 100 ppm. Our filter still maintained high performance at 500 ppm, although the saturation effect on the binding sites was evident. Mechanistic studies revealed the lead deposition on yeast cells and filter surfaces, confirming the role of heavy metal removal as bioremediation.

Overall, strain-specific metal removal optimization and the implementation of the learning to assemble a consortium were showcased in our study. The augmented filter provides an excellent tool for lead removal from water samples and opens up avenues for wastewater remediation and industrial applications in the future. Our study lays a robust foundation for further advanced studies on the potential utilization of native microbial consortia from the river Cauvery and their implementation in industrial, agricultural, and wastewater remediation purposes.

## Data Availability

Publicly available datasets were analyzed in this study. This data can be found here https://www.ncbi.nlm.nih.gov/nuccore/, accession numbers: Candida tropicalis-R1N6 (PP972331), Candida tropicalis-R2N1 (PP917747), Candida tropicalis-R2N4 (PP917748), Pichia kudriavzevii-R1N8 (PP972332), and Clavispora lusitaniae-R4N2 (PP972335).
