# Supplementary material for: From consortium design to bioaugmented filters: scalable yeast-based strategies for lead remediation in water systems
Source: Front Microbiol. 2025 Sep 10;16:1647398. doi: 10.3389/fmicb.2025.1647398 (PMC12457418; doi:10.3389/fmicb.2025.1647398)

**From Consortium Design to Bioaugmented Filters: Scalable Yeast-Based Strategies for Lead Remediation in Water Systems**

**Nikita Gupta^1, 2^ and Sathiavelu Arunachalam^2*^**

^1^ School of BioSciences and Technology, VIT, Vellore-632014, Tamil Nadu, India.

^2^ VIT School of Agricultural Innovation and Advanced Learning, VIT, Vellore-632014, Tamil Nadu, India

*** Correspondence:**

Sathiavelu Arunachalam^*^

asathiavelu@vit.ac.in

**Supplementary Data**

**Table S1:** Process variables in range form for Pb2+ ions biosorption using *Pichia kudriavzevii, Candida Topicalis* and *Clavispora lusitaniae*

| **Factor** | **Parameters** | **Units** | **Range and Levels** | |
| --- | --- | --- | --- | --- |
|  |  |  | **Lower** | **Upper** |
| 1 | pH | -- | 3 | 7 |
| 2 | Biomass | g | 0.5 | 2 |
| 3 | Pb Concentration | mg/L | 100 | 200 |

**Table S2:** ANOVA of Pb2+ ions biosorption using *Pichia kudriavzevii* quadratic model

| **Source** | **Sum of Squares** | **df** | **Mean Square** | **F-value** | **p-value** |  |
| --- | --- | --- | --- | --- | --- | --- |
| **Model** | 18732.14 | 9 | 2081.35 | 12.90 | 0.0028 | significant |
| A-pH | 7074.49 | 1 | 7074.49 | 43.85 | 0.0006 |  |
| B-Biomass | 3063.00 | 1 | 3063.00 | 18.98 | 0.0048 |  |
| C-Pb Conc | 73.64 | 1 | 73.64 | 0.4565 | 0.5245 |  |
| AB | 836.25 | 1 | 836.25 | 5.18 | 0.0631 |  |
| AC | 703.83 | 1 | 703.83 | 4.36 | 0.0818 |  |
| BC | 115.43 | 1 | 115.43 | 0.7154 | 0.4301 |  |
| A² | 1869.79 | 1 | 1869.79 | 11.59 | 0.0144 |  |
| B² | 2523.16 | 1 | 2523.16 | 15.64 | 0.0075 |  |
| C² | 677.89 | 1 | 677.89 | 4.20 | 0.0863 |  |
| **Residual** | 968.03 | 6 | 161.34 |  |  |  |
| Lack of Fit | 717.30 | 5 | 143.46 | 0.5722 | 0.7566 | not significant |
| Pure Error | 250.73 | 1 | 250.73 |  |  |  |
| **Cor Total** | 19700.17 | 15 |  |  |  |  |

**
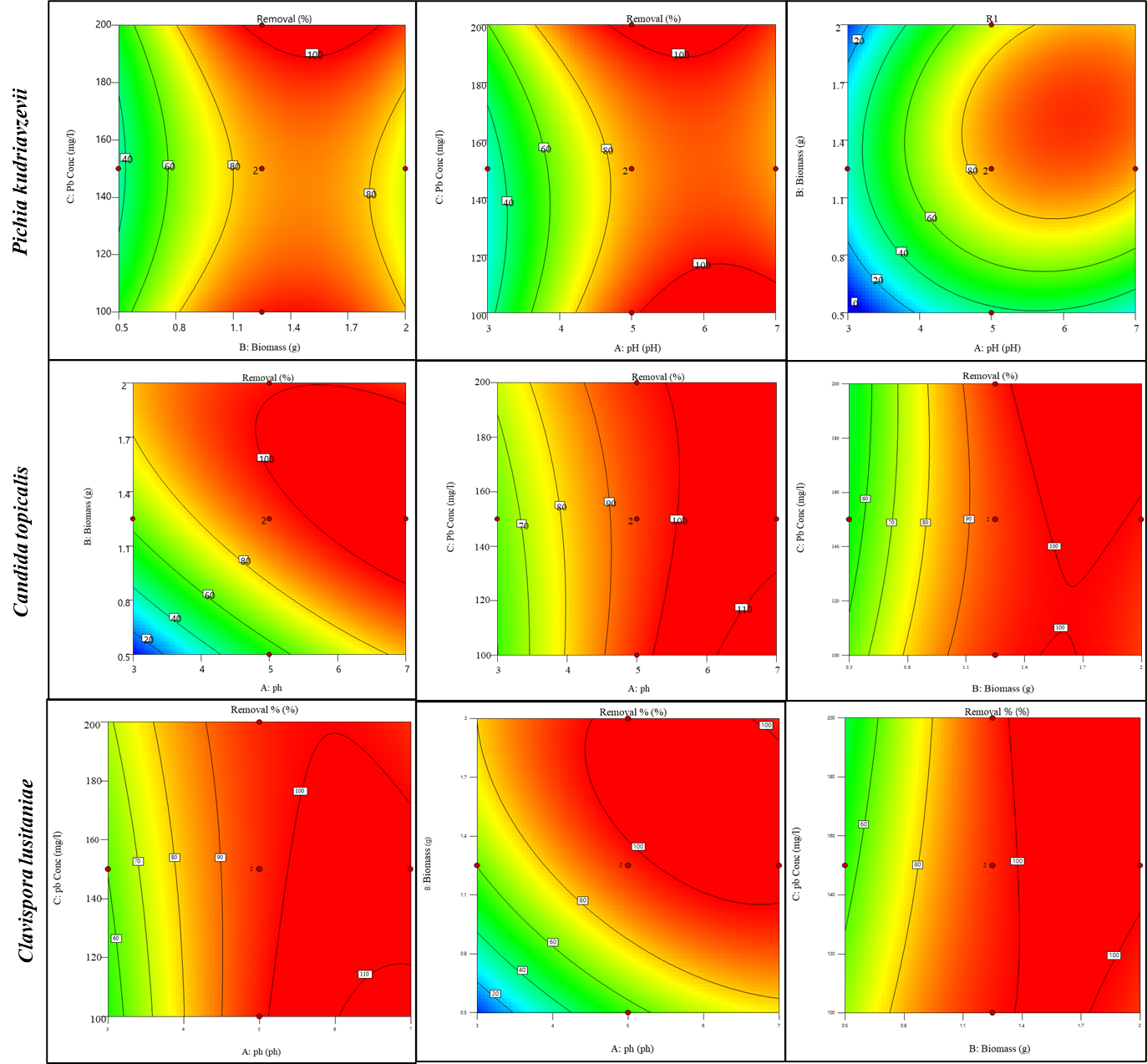
**

**Figure S1:** 2-D Response Surface plots of Pb2+ ions biosorption using *Pichia kudriavzevii, Candida topicalis, and Clavispora lusitaniae*

**
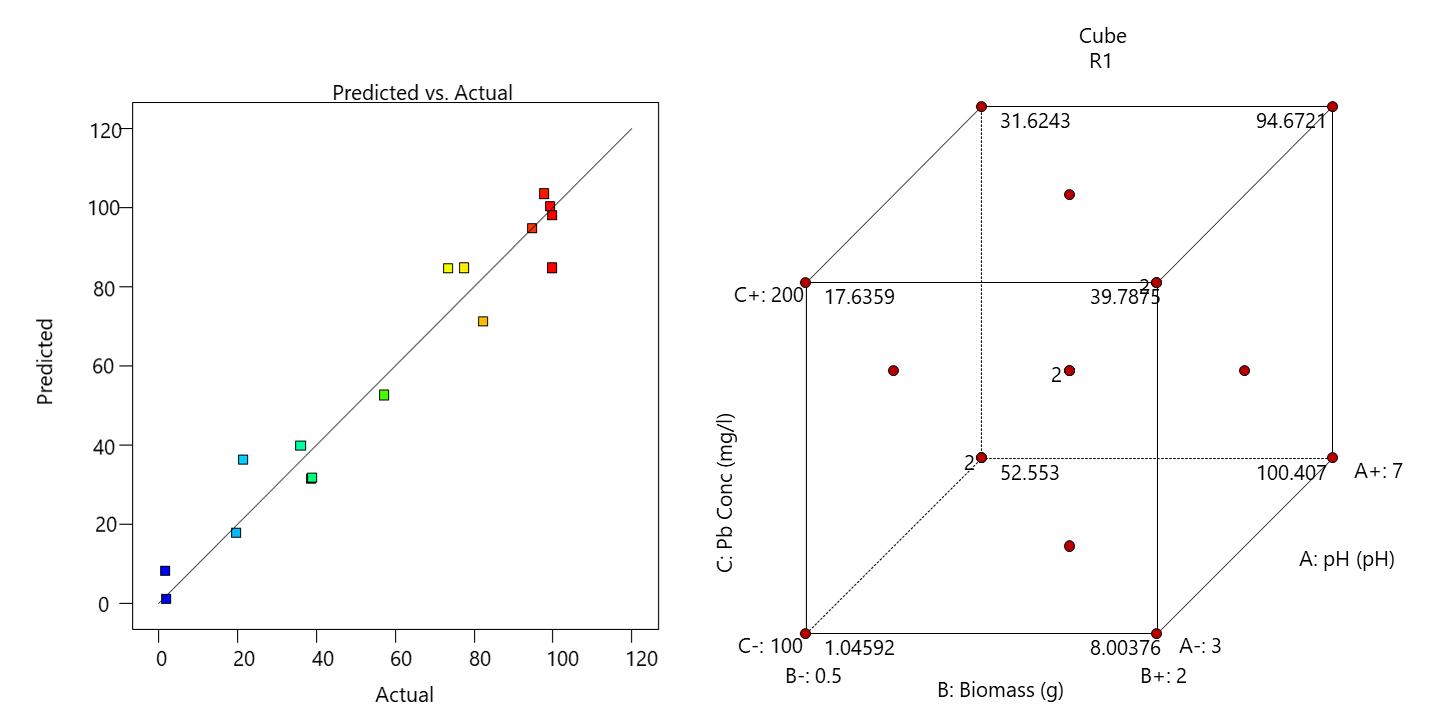
**

**Figure S2:** Normal probability plot (A) and Cube plot (B) for biosorption (%) of Pb2+ ions

**Table S3:** ANOVA of Pb2+ ions biosorption using *Candida topicalis* quadratic model

| **Source** | **Sum of Squares** | **df** | **Mean Square** | **F-value** | **p-value** |  |
| --- | --- | --- | --- | --- | --- | --- |
| **Model** | 14134.84 | 9 | 1570.54 | 43.21 | < 0.0001 | **significant** |
| A-ph | 4755.74 | 1 | 4755.74 | 130.86 | < 0.0001 |  |
| B-Biomass | 4916.12 | 1 | 4916.12 | 135.27 | < 0.0001 |  |
| C-Pb Conc | 0.1626 | 1 | 0.1626 | 0.0045 | 0.9488 |  |
| AB | 1929.14 | 1 | 1929.14 | 53.08 | 0.0003 |  |
| AC | 311.75 | 1 | 311.75 | 8.58 | 0.0263 |  |
| BC | 354.71 | 1 | 354.71 | 9.76 | 0.0205 |  |
| A² | 216.08 | 1 | 216.08 | 5.95 | 0.0506 |  |
| B² | 851.18 | 1 | 851.18 | 23.42 | 0.0029 |  |
| C² | 17.76 | 1 | 17.76 | 0.4888 | 0.5106 |  |
| **Residual** | 218.06 | 6 | 36.34 |  |  |  |
| Lack of Fit | 214.75 | 5 | 42.95 | 12.97 | 0.2076 | **not significant** |
| Pure Error | 3.31 | 1 | 3.31 |  |  |  |
| **Cor Total** | 14352.90 | 15 |  |  |  |  |

**
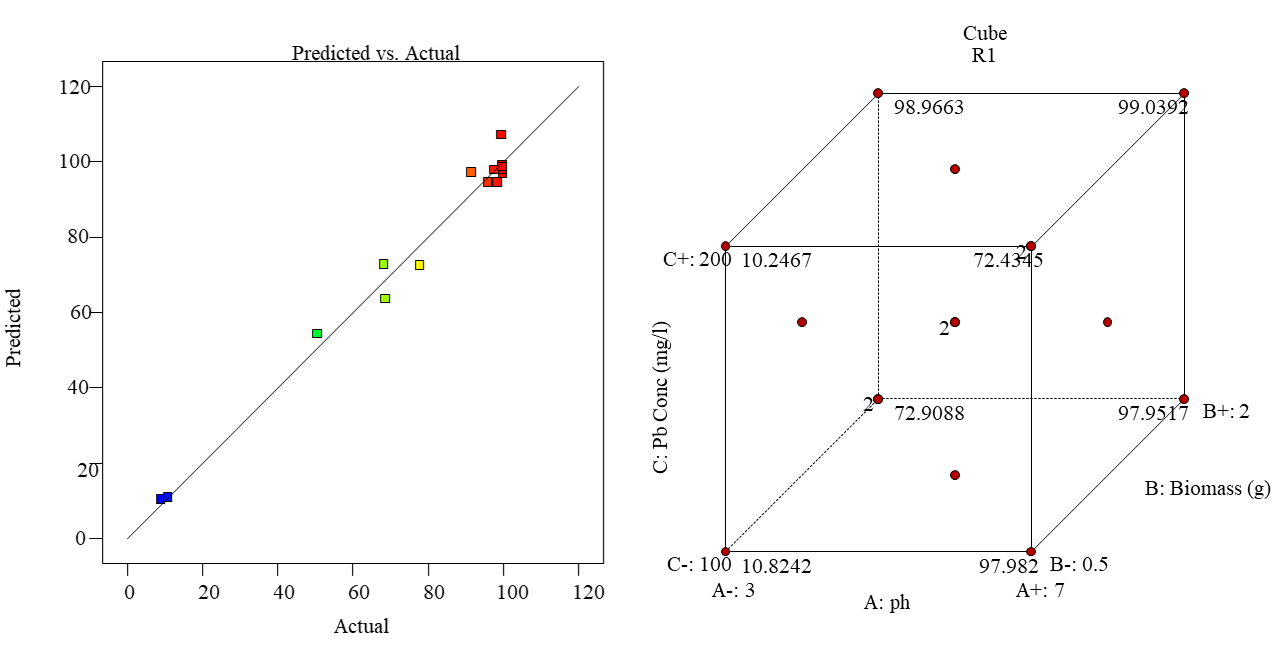
**

**Figure S3:** Normal probability plot (A) and Cube plot (B) for biosorption (%) of Pb2+ ions

**Table S4:** ANOVA of Pb2+ ions biosorption using *Clavispora lusitaniae* quadratic model

| **Source** | **Sum of Squares** | **df** | **Mean Square** | **F-value** | **p-value** |  |
| --- | --- | --- | --- | --- | --- | --- |
| **Model** | 15821.21 | 9 | 1757.91 | 152.25 | < 0.0001 | significant |
| A-ph | 4711.47 | 1 | 4711.47 | 408.05 | < 0.0001 |  |
| B-Biomass | 5680.48 | 1 | 5680.48 | 491.97 | < 0.0001 |  |
| C-pb Conc | 7.27 | 1 | 7.27 | 0.6294 | 0.4578 |  |
| AB | 1434.40 | 1 | 1434.40 | 124.23 | < 0.0001 |  |
| AC | 529.22 | 1 | 529.22 | 45.83 | 0.0005 |  |
| BC | 760.26 | 1 | 760.26 | 65.84 | 0.0002 |  |
| A² | 545.13 | 1 | 545.13 | 47.21 | 0.0005 |  |
| B² | 807.81 | 1 | 807.81 | 69.96 | 0.0002 |  |
| C² | 2.61 | 1 | 2.61 | 0.2260 | 0.6513 |  |
| **Residual** | 69.28 | 6 | 11.55 |  |  |  |
| Lack of Fit | 67.14 | 5 | 13.43 | 6.29 | 0.2935 | not significant |
| Pure Error | 2.14 | 1 | 2.14 |  |  |  |
| **Cor Total** | 15890.49 | 15 |  |  |  |  |

**
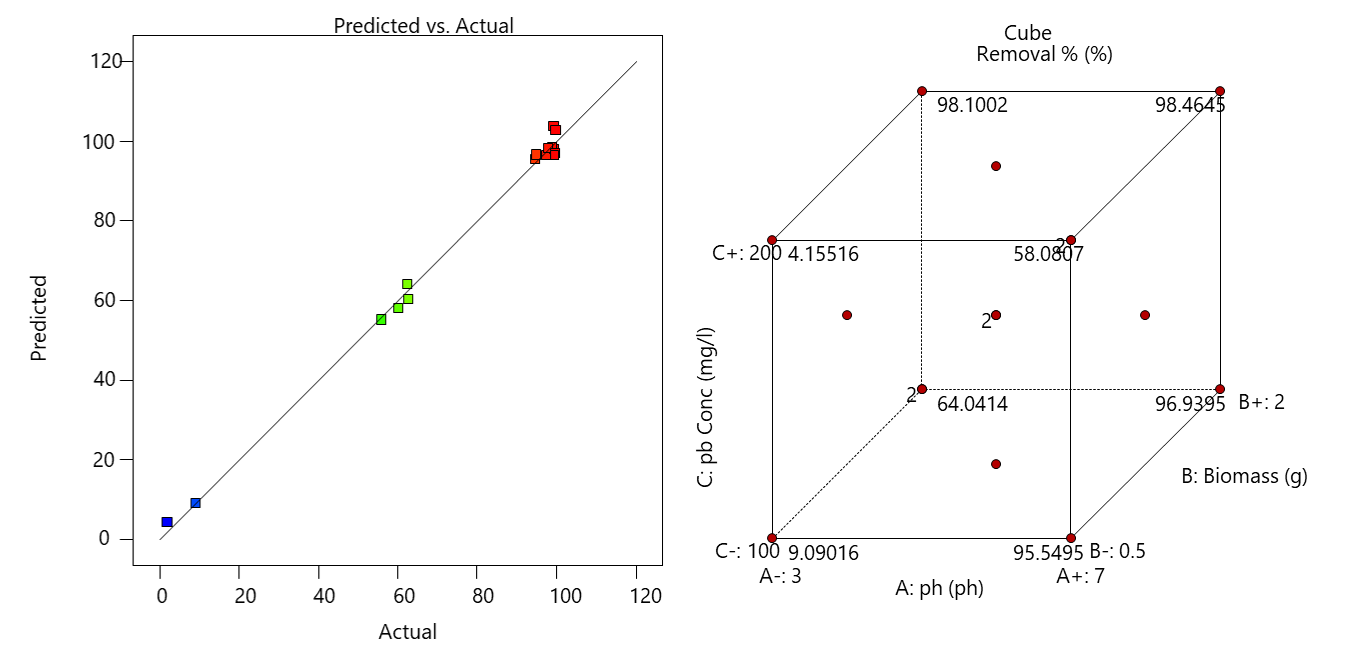
**

**Figure S4** Normal probability plot (A) and Cube plot (B) for biosorption (%) of Pb2+ ions

**Table S5:** Characteristics of Alginate Yeast Gel Slab or Bioaugmented Filter

| **Characteristics** | |
| --- | --- |
| Diameter | 47 mm |
| Thickness | approximately 2.31 mm |
| Volume | 4 mL |
| Gelation time | 30 Min |
| Clarity | Creamy White |

**Figure S5:** A Bio-augmented filter


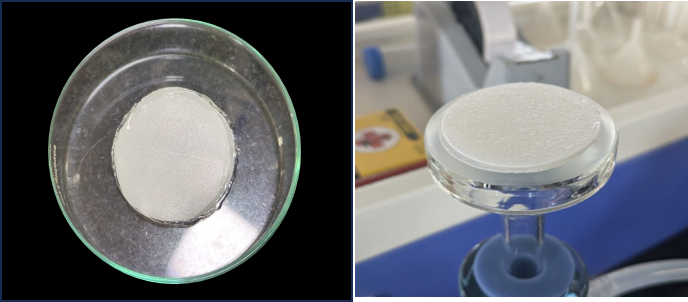

Supplement: Supplementary file 1 [file Supplementary_file_1.docx]
